# Multilevel analysis of site, implant, and patient-level factors with peri-implant bleeding on probing: a cross sectional study

**DOI:** 10.1186/s40729-021-00315-0

**Published:** 2021-06-10

**Authors:** Sunil Kumar Nettemu, Sowmya Nettem, Vijendra Pal Singh, Sheila Shirley William, Shargunan Selvanthan Gunasekaran, Malathi Krisnan, Adinegara Lutfi Abas

**Affiliations:** 1 Manipal University College Malaysia, Melaka City, Malaysia; 2 Klinik Pergigian Senawang, Persiaran Senawang 2, 70450 Seremban, Malaysia; 3 Klinik Kesihatan Daerah Manjung-Seri Manjung, 32000 Perak, Malaysia; 4 Klinik Pergigian Kangar, 01000 Kangar, Perlis Malaysia

**Keywords:** Bleeding on probing, Gingival biotype, Peri-implant diseases

## Abstract

**Aim:**

This study was to evaluate the association between peri-implant bleeding on probing in peri-implant diseases and its association with multilevel factors (site specific factors, implant factors, and patient level factors).

**Methodology:**

A cross-sectional study involved consented adult patients with ≥ 1 dental implant. Two calibrated operators examined the patients. BoP was outcome variable and peri-implant gingival biotype was principal predictor variable. The effects of site, implant, and patient level factors on BoP were assessed using a multilevel logistic regression model.

**Results:**

Eighty patients for a total of 119 implants and 714 sites were included in the study. Bleeding on probing was observed in 42 implants (35.29%) with a significant higher risk observed in presence of gingival recession, thin peri-implant gingival biotype, duration of implant placement, smokers, and male patients.

**Conclusion:**

Peri-implant bleeding on probing was associated with site specific, implant, and patient level factors.

**Supplementary Information:**

The online version contains supplementary material available at 10.1186/s40729-021-00315-0.

## Introduction

Peri-implant diseases are inflammatory conditions affecting the soft and hard tissues around dental implants. Peri-implant disease is a serious problem that plagues today’s dentistry in terms of therapy and epidemiology. Peri-implant mucositis is the presence of inflammation of the peri-implant mucosa without signs of loss of bone support, while peri-implantitis, in addition to inflammation of the mucosa, is characterized by a loss of bone support [1, 2]. Peri-implantitis was often defined by the incidence of peri-implant probing depth ≥ 5 mm associated with bleeding on probing (BoP) and/or suppuration and radiographic images of bone loss [3, 4]. Cases where the radiographs did not confirm the peri-implant bone loss were diagnosed as peri-implant mucositis [5].

The joint use of probing depth, radiographic bone loss, and BoP was frequently implemented in the clinical diagnosis of peri-implant disease [6]. There are, however, notable exceptions. In the 7th European Workshop on Periodontology, peri-implantitis was characterized by changes in the level of the crestal bone in conjunction with BoP with or without concomitant deepening of peri-implant pockets [7].

**Fig. 1:**
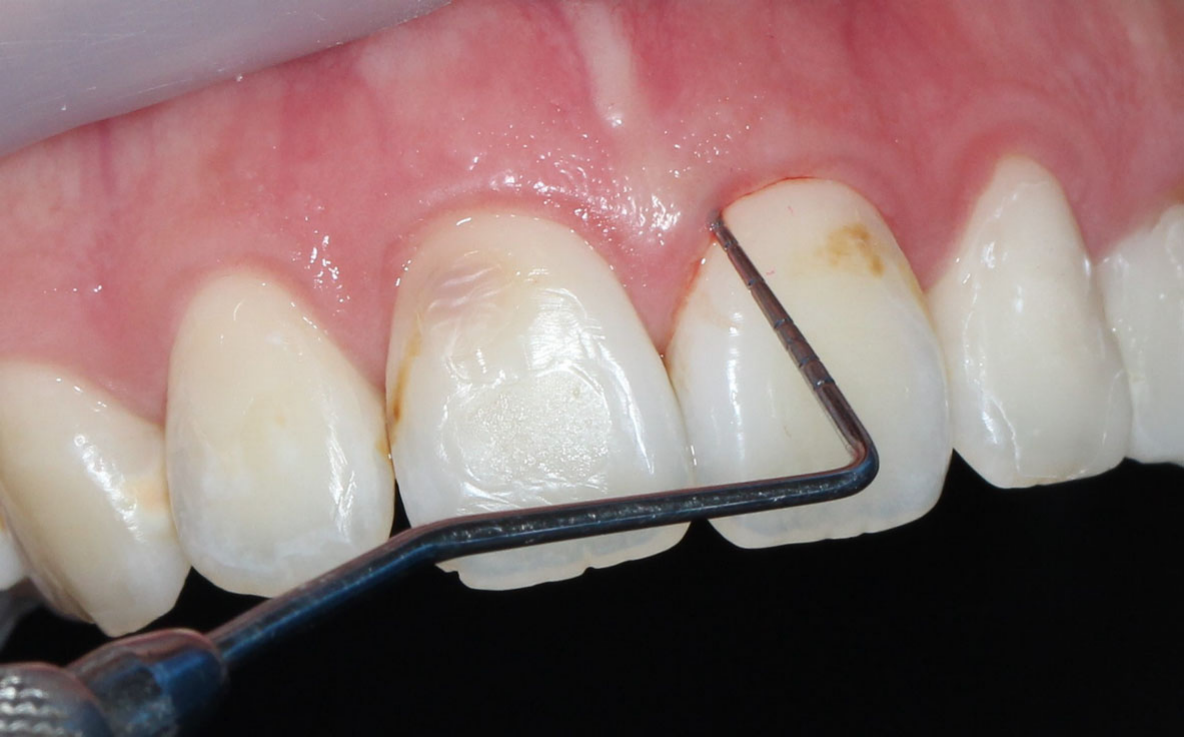
Peri-implant bleeding on probing

Peri-implant bleeding on probing (Fig. 1) has a decisive value in the classification and diagnosis of peri-implant disease. Bleeding on probing is considered as key clinical measure to distinguish between peri-implant health and disease [8]. The BoP measurement can change the diagnosis from a healthy implant to mucositis, as well as from an unclassified situation (probing depth+, BoP, Bone Loss+) to peri-implantitis [9].

Recently, a study evaluated a retrospective observational study to determine BoP associated risk factors for dental implant [10]. They found that the rate to be BoP+ for a site with PD = 3 was 18% and the odds ratio increased by 1.98 for each 1 mm increment in PD [10]. In addition, a significantly higher risk for BoP+ was observed for interproximal vs. approximal surface, posterior teeth vs. anterior teeth, and female vs. male, while a significantly lower risk was observed for smokers vs. non-smokers [10].

Furthermore, a cross-sectional study was done to evaluate the association between peri-implant BoP and probing depth. Other factors regarding patients, implants, and sites were taken into consideration. The final model comprehended only the probing depth and the site position. The odds ratio of a site to be BoP increased by 1.81 for each 1 mm increment in probing pocket depth. A significant higher risk was observed also for interproximal vs. approximal implant surfaces. Other considered variables at patient, implant, or site level were all not significant when considered in conjunction with probing depth and site position. Though various studies has been done to evaluate the association of peri-implant bleeding on probing with multilevel factors [11], no specific studies have been to evaluate the association of peri-implant bleeding on probing with peri-implant gingival biotype in respect to its functional aspects.

Gingival biotypes are the critical factor that determines the outcome of an implant placement. The term gingival biotypes used to describe the thickness of gingiva in facio-palatal dimension [12]. According to Ochsenbein and Ross, there were two types of gingival morphology, namely scalloped and thin or flat and thick gingiva. Studies have proven that peri-implant diseases are more likely to occur in patient with thin biotypes as they have a compromised soft tissue response following surgical treatment [12]. Cochran stated a need of 3 mm of peri-implant mucosa is needed for a stable epithelial, connective tissue attachment. The gingival thickness affects the treatment outcome possibly because of the difference in amount of blood supply of the underlying bone and susceptibility to resorption [13].

**Fig. 2:**
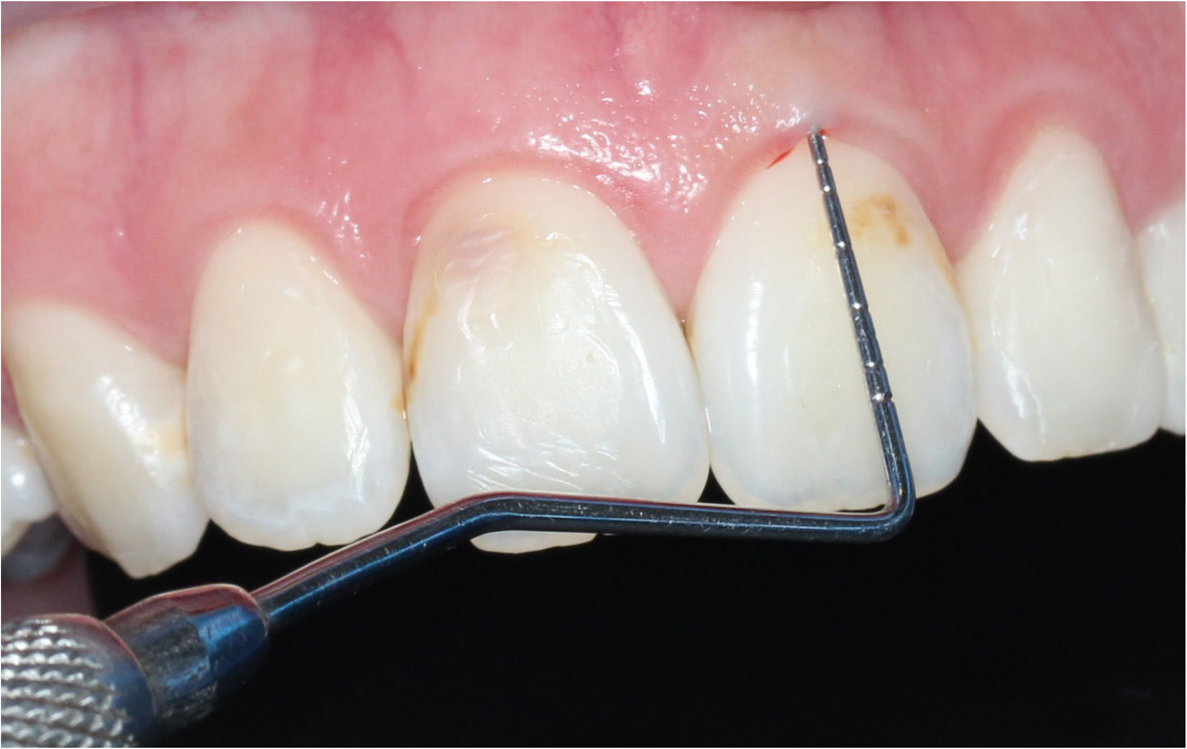
Probe not visible through sulcus (thick gingival biotype)

**Fig. 3:**
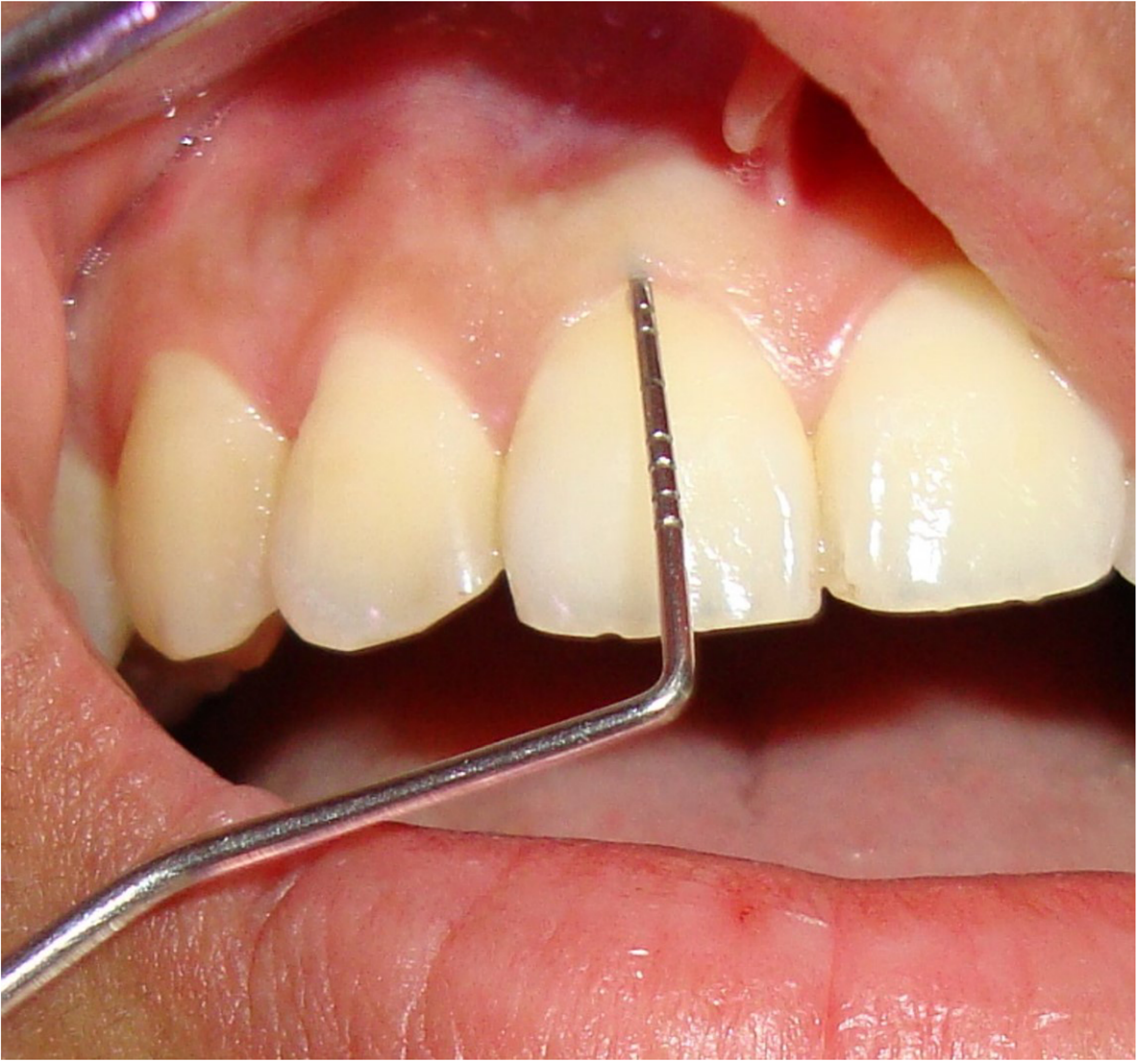
Probe visible through sulcus (thin gingival biotype)

Many invasive and non-invasive methods used to measure gingival biotypes. Transgingival probing is a traditional invasive method which has limited application in clinic. Instead of this, another method based on the translucency of the periodontal probe through the gingival margin upon inspection is widely used and is taken as a simple method with excellent repeatability (Figs. 2 and 3). Ultrasonic measurement and cone beam computerized tomography (CBCT) are also non-invasive methods, but specific devices are needed for these assessments [13].

Recently, a study was done to evaluate the role gingival biotypes in esthetic success of dental implant by Mahdi Kadkhodazade but not in terms of the functional aspects [14]. Thus, this study was carried out to determine the functional aspects of gingival biotypes in determining peri-implant diseases. In addition, Vandana and Savitha, in a 2005 [15] study in humans, and Kyllar and Witter, in a 2008 study in dogs, demonstrated that gingival thickness varies by sex and age as well as dental arch forms. However, to the best of our knowledge, there were no studies done to determine the association of peri-implant bleeding on probing in respect to different ethnicities in Malaysian population.

Hence, the objective of this study was to evaluate the association between peri-implant bleeding on probing in peri-implant diseases and its association with multilevel factors which include site specific factors (gingival recession, bone loss, pocket depth, and peri-implant gingival biotype), implant level factors ( position and duration of implants), and patient level factors (age, gender, ethnicity, smoking, and systemic diseases) which highlights on the functional aspect of peri-implant gingival biotype.

## Material and methods

### Study design and setting

It was a cross-sectional study involving consented patients who have received dental implants therapy at Faculty of Dentistry, Melaka-Manipal Medical College, Malaysia, between March 2011 and March 2017. The study was approved by the Ethics and Research Committee of Faculty of Dentistry, Melaka-Manipal Medical College, Malaysia and conducted in accordance with the Helsinki Declaration of 1975, as revised in 2000. In this study, a purposive sampling was followed. The reference population for this study included all residents in Melaka Tengah and the source population included all patients who received dental implant treatment at Faculty of Dentistry, Melaka-Manipal Medical College, Malaysia.

Inclusion criteria was as follows:

1. Participants who were 18 years or older in age.
2. Patients of both male and female genders.
3. Patients with presence of at least one implant which has been functionally loaded for a minimum of 1 year.
4. Patients with implant supported fixed prosthesis.
5. Patients with previous radiograph of the implant site taken at the time of placement.
6. Patients providing written informed consent for taking radiograph currently for the purpose of the study and which will serve as a post-operative radiograph to check for implant health and peri-implant bone status.
7. Smokers and non-smokers
8. Patients with systemic illness were included

Exclusion criteria was as follows:

1. Dental implants with cemented restorations
2. Patients irradiated in head and neck area.
3. Drug abusers
4. Pregnant and lactating mothers
5. Patients who have undergone immunosuppressive therapy in the previous 5 years.

### Variables and measurements

Two operators who were previously calibrated in peri-implant measures and diagnosis examined the patients. Before clinical examination was done, patients were explained about the procedure and consent was obtained. Following information was recorded for each patient: site-specific factors (peri-implant gingival biotype, probing depth, gingival recession and bone loss), patient factors (age, gender, ethnicity, smoking habit, and systemic status), implant factors (position of implant and duration of implant), and the outcome (BOP).

The outcome variable was peri-implant bleeding on probing and the principal predictor variable include peri-implant gingival biotype. Other predictor variables at patient level were as follows: age, gender, ethnicity, smoking habit, systemic status while the predictor variable at implant level include position and duration of implants. Other predictor variables at site-specific level were as follows: bleeding on probing, pocket depth and bone loss, and gingival recession. Probing depth was measured at six sites per implant (mesio-buccal, mid-buccal, disto-buccal, mesio-lingual, mid-lingual, disto-lingual).

Gentle probing was applied using PCPUNC15 probe. Bleeding on probing, gingival recession was measured at per implant site. Gingival biotype was categorized as thin or thick based on the probe translucency. Pre-operative radiograph and radiograph taken at time of recall was compared and bone loss is classified as present or absent. The site of implant placement was categorized as upper right posterior, upper left posterior, upper anterior, lower left posterior, lower right posterior, and lower anterior. Patients history of systemic diseases were limited to diabetes and hypertension as they play a role in peri-implantitis. The examiner (1 or 2) was considered as a potential confounding variable.

### Bias

The two examiners were previously aligned and calibrated in peri-implant measures and diagnosis [9]. The agreement between the two examiners regarding the peri-implant diseases was 0.63 (kappa statistics), and regarding the peri-implant probing depth was 0.57 (intraclass correlation coefficient) [9]. The influence of the examiner was considered in the statistical model as a confounding variable.

### Statistical analysis

Descriptive statistics were performed considering frequency and percentage for qualitative variables and mean and standard deviation for quantitative variables. Multilevel logistic regression models were performed using three levels: patient level, implant level, and site level. Six sites per implant were considered. The outcome variable was bleeding on probing applied as a dichotomous mode (yes or no). The primary predictor variable was gingival biotype. The other covariates were tested in bivariate analyses. The covariates found significant were included in a model. The impact of each factor on bleeding on probing was expressed as a 95% confidence interval (95% CI) and an odds ratio (OR) was provided. The accuracy of the final model was calculated using threshold the prediction probability of BoP+ of 0.5. Multilevel analysis was performed using IBM SPSS Statistics trial version (Additional file 1).

## Results

### Participants and descriptive data

Eighty patients for a total of 119 implants and 714 sites were included in the study [16]. The mean age was 56.35 years (standard deviation 10.8, range 23–78 years). Forty-two patients (52.5%) were female, 52 patients (65.0%) were Chinese, 16 patients (20.0%) were Malay, and 12 patients (15.0%) were Indian. Fifteen patients (18.75%) were hypertensive, 11 patients (13.75%) were diabetic, 3 patients (3.75%) were known asthmatic, 6 patients (7.5%) were smokers, and 32 patients (40.0%) were taking prescription drugs.

One hundred ten (92.43%) of the implants were situated in posterior area and were inserted 1.7 (SD 0.87) years before current examination of the implants.

Bleeding on probing was observed in 42 implants (35.29 %), probing depth was 1.2 (SD 1.37) mm and recession seen in 27 implants (22.69%), gingival biotype were thin in 29 implants (24.73 %) and bone loss observed in 13 implants (10.92 %)

### Outcome data

The bivariate analyses referring to bleeding on probing are presented in Table 1. Significant variables were the systemic disease (yes), smoking (yes), and age for patient level factor. Implants in lower right posterior, upper left posterior, and upper right anterior for implant level factor. Site level factors on recession (present), bone loss (present), and probing depth were significant.

**Table 1:** Results of descriptive statistics

**Site level factors—level 1**	***n*(percentage, %)**
Peri-implant bleeding on probing	42 (35.29)
Gingival recession (present)	27 (22.69)
Gingival biotype (thin)	29 (24.37)
Bone loss (present)	13 (10.92)
**Implant level factors—level 2**	***n*(percentage, %)**
**Duration (in months)**	
12–23	52 (43.70)
24–35	59 (49.60)
36–47	3 (2.51)
48–59	3 (2.51)
60–71	0
72–83	2 (1.68)
**Position**	
Anterior	9 (7.57)
Posterior	110 (92.43)
**Patient level factors—level 3**	***n*(percentage, %)**
**Gender**	
Female	42 (52.5)
Male	38 (47.5)
**Ethnicity**	
Chinese	52 (65.0)
Malay	16(20.0)
Indian	12(15.0)
**Age**	
20–34	4 (5.0)
35–49	19 (23.75)
50–64	45 (56.25)
65–79	12 (15.0)
**Systemic disease**	
Hypertension	15 (18.75)
Diabetes mellitus	11 (13.75)
Asthma	3 (3.75)
**Smoking habit** (smokers)	6 (7.5)

### Main results

The number of natural teeth, the number of implants at patient level, the site position (interproximal vs. approximal), and probing depth at site level were inserted in a new model. The number of natural teeth and the number of implants were no longer significant and were deleted from the model.

The final model comprehended only the probing depth, bone loss, gingival biotype, and gingival recession (Table 2). The odds ratio increased by 1.81 (95% CI from 1.47 to 2.23; *P* < 0.0001) for each 1 mm increment in probing pocket depth. A significant higher risk was observed also for interproximal vs. approximal surfaces (OR = 1.55; 95% CI from 1.02 to 2.36; *P* = 0.0402). The probability for a site to be BoP+ in relation to pocket depth and site position is illustrated in Table 2. BoP+ is about 30–40% for a pocket depth of 3 mm, but it is over 80% for a pocket depth of 7 mm. With equal pocket depths, the probability for a site to bleed on probing (BoP+) is greater for interproximal sites with respect to approximal sites. The prediction accuracy of the model was 66%.

**Table 2:** Results of bivariate analysis referring to bleeding on probing

Variable	Odds ratio	95% CI	p value
**Gingival biotype**			
*Thick*	1.000	–	–
*Thin*	1.467	1.040–2.069	0.029
**Gingival recession**			
*Absent*	1.000	–	–
*Present*	2.601	1.817–3.722	< 0.001
**Bone loss**			
*Absent*	1.000	–	–
*Present*	1.778	1.108–2.852	0.017
**Pocket depth**	1.975	1.713–2.276	< 0.001
**Duration of implant (months)**	0.981	0.966–0.997	0.021
**Position of implant**			
*Upper right posterior*	1.000	–	–
*Lower left anterior*	0.000	0.000	0.999
*Lower left posterior*	1.010	0.636–1.605	0.966
*Lower right anterior*	0.000	0.000	0.999
*Lower right posterior*	0.584	0.352–0.969	0.037
*Upper left anterior*	0.000	0.000	0.999
*Upper left posterior*	2.571	1.363–4.850	0.004
*Upper right anterior*	0.643	0.288–1.433	0.280
**Gender**			
*Female*	1.000	–	–
*Male*	1.357	1.006–1.832	0.046
**Ethnicity**			
*Chinese*	1.000	–	–
*Indian*	1.581	1.039–2.405	0.032
*Malay*	1.185	0.796–1.765	0.402
**Systemic disease**	1.327	0.982–1.793	0.065
**Smokers**	0.288	0.151–0.549	< 0.001
**Age**	1.027	1.012–1.042	< 0.001

Table 3 shows the results of multilevel logistic regression (generalized linear) after backward stepwise logistic regression (conditional) was performed. The final model showed significant findings for gender, smoking, and duration of dental implants. The odds ratio for females showed that female gender was 11.599 times more likely to develop peri-implant BoP. No smoking protects from bleeding on probing as odds ratio for non-smoking which was 1–0.003 meant 99.7% reduction in bleeding on probing among non-smokers in comparison to smokers. Longer duration after dental implant placement and functional loading was found to be less likely to bleed with 11.3% reduction in bleeding among patients with longer duration of implants for every 1 year of duration. All the four variables (gingival recession, gingival biotype, pocket depth, and bone loss) were inserted into the final model and subjected to stepwise-logistic regression analysis. Pocket depth and bone level were noted to be no longer significant while gingival recession and gingival biotype were still noted to be positively significant with peri-implant bleeding on probing.

**Table 3:** Results of multilevel logistic regression (generalized linear) after backward stepwise logistic regression (conditional) was performed

Model term	Coefficient	Std. error	t.	Sig.	Exp (coefficient)	95% confidence interval for exp (coefficient)	
						Lower Upper	
**Intercept**	− 21.850	7,060.389	− 0.003	.998	0.000	0.000	–
**Gender (males)**	2.451	1.238	1.980	.048	11.599	1.020	131.877
**Smoking (smokers)**	− 5.771	2.654	− 2.174	.030	0.003	0.000	0.571
**Duration**	− 0.120	0.056	− 2.153	.032	0.887	0.795	0.990
**Gingival biotype (thin)**	3.410	1.066	3.199	.001	30.273	3.733	245.499
**Gingival recession (present)**	4.100	0.950	4.314	.000	60.316	9.336	389.673

## Discussion

The objective of this study was to evaluate the association between peri-implant bleeding on probing in peri-implant diseases and its association with multilevel factors which include site specific factors, implant level factors and patient level factors. A significant higher risk for bleeding on probing was noticed in patients with systemic diseases which include diabetes and hypertension, patients with habits of smoking, and with increasing age. Among the implant level factors, there is higher risk of peri-implant bleeding on probing seen in relation to lower right posterior, upper left posteriors, and upper anteriors.

A similar study was done by Carcuaco and Janssonl which concluded the percentage of implants with peri-implantitis was significantly increased for smokers compared to non-smokers (*p* = 0.04). In the group of non-smokers, 64% of the implants had the diagnosis peri-implantitis, while the corresponding relative frequency for smokers was 78%. A majority of the individuals had a plaque index and bleeding on probing index > 50%.

In the study on natural teeth, a significant higher risk was also observed for posterior teeth vs. anterior teeth (OR = 1.15), females vs. males (OR = 1.17), while a significant lower risk was observed for smokers vs. non-smokers (OR = 0.83) [10]. These variables were not significant in this study where implants were concerned. The differences could be due to the low number of selected categories in this study; for example, the smokers were only eight (15%)

Based on the European Review for Medical and Pharmacological Science, a meta-analysis reported a 50% higher risk of detecting peri-implantitis in subjects with diabetes/hyperglycemia compared to non-diabetes patients (RR = 1.46; 95% CI 1.21–1.77 and OR = 1.89; 95% CI 1.31–2.46; *p* < .001).

For the site level factors, a significant higher risk was observed in patients with a thin gingival biotype, gingival recession, bone loss and increasing pocket depth. The results are very similar to the referring study on natural teeth [10]. They had found an odds ratio of 1.98 and 1.91 for probing depth for the analysis with only one and with all predictors, respectively [10]. A deep pocket may be at a higher risk to bleed upon probing due to the quantitative and/or qualitative aspects of the subgingival bacterial challenge [17, 18]. On the other hand, when a site presents a considerable probing depth (e.g., 7 mm), the percentage of sites with no bleeding is very low (nearly 10%). This situation is evidently so rare that it has not been classified. In fact, an implant with a pocket depth of 7 mm with bone loss, but with no BOP, cannot be considered healthy nor affected by peri-implantitis or mucositis [9].

In a 2013 review study on 11 articles, Lin et al. stated that the presence of keratinized mucosa around implant was associated with less attachment loss and gingival marginal recession whereas Bengazi et al. reported that ST loss around implants can merely be the result of tissue regeneration for the stabilization of biologic width by the peri-implant mucosa. The difference in results may be due to the effect of confounding factors, such as differing follow-up times, implant position, quality of soft tissue and hard tissue and oral hygiene standards among the studies.

The main principal predictor variable (gingival biotype) plays an important role in the gap statement of this study. A thin gingival biotype has a higher risk in peri-implant bleeding on probing. This is probably due to the reason thin gingival tissue is associated with a thin band of the keratinized tissue, scalloped gingival contour suggestive of thin bony architecture and is more sensitive to inflammation and trauma thus more prone to BOP. Based on the results reported by Sammartino et al. and Belser et al., the presence of thin peri-implant soft tissue increases the risk of gingival recession and subsequent exposure of the metal margin of the implant prosthesis.

Other patient level and implant level factors were not taken into consideration as they were not significant. The focus of this study was on the relationship between peri-implant gingival biotype and BoP and the power was tailored to this relationship. In conclusion, the result of this study indicates that the probability of an implant site to bleed upon probing was associated with peri-implant gingival biotype noting that patients with increasing age, smoking habit, and presence of systemic diseases tend to have higher chances of bleeding upon probing and have a higher risk of bleeding on probing in patients with bone loss, gingival recession and increase pocket depth.

## Conclusion

There is a statistically significant association between gingival recession and mainly thin gingival biotype with peri-implant BoP. Moreover, there is a significant association between duration of implant placement, males, and smokers with peri-implant BoP. Since these factors contribute to the occurrence of peri-implant diseases, they should be taken into consideration during treatment planning so that more appropriate strategies for periodontal management may be developed, resulting in more predictable treatment outcomes

## Supplementary Information

**Additional file 1: Figure 1.** Diagram 1 shows Process of Statistical Analysis using IBM SPSS Software trial version.

## Data Availability

Not applicable.

## Abbreviations

BoP: Bleeding on probing
BoP +: Bleeding on probing present
PD: Probing depth
CBCT: Cone beam computerized tomography
SD: Standard deviation
CI: Confidence interval
OR: Odds ratio
RR: Relative risk
PCP-UNC 15: University of North Carolina probe
