# Supplementary figures and images for: Multilevel analysis of site, implant, and patient-level factors with peri-implant bleeding on probing: a cross sectional study
Source: Int J Implant Dent. 2021 Jun 10;7:77. doi: 10.1186/s40729-021-00315-0 (PMC8190223; doi:10.1186/s40729-021-00315-0)

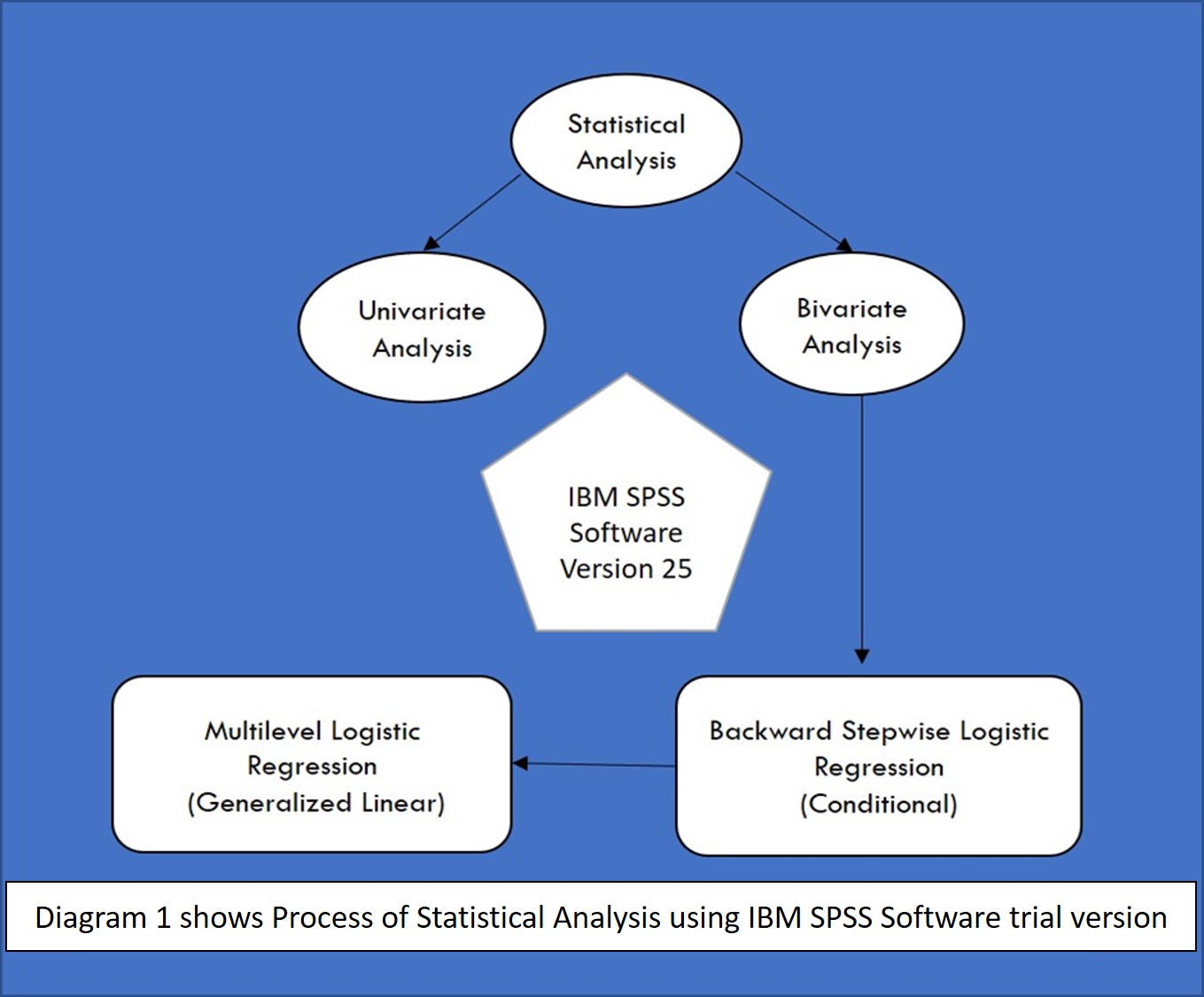

Supplement: Supplementary file 1 — Additional file 1: Figure 1. Diagram 1 shows Process of Statistical Analysis using IBM SPSS Software trial version. [file 40729_2021_315_MOESM1_ESM.jpg]
